# pH-triggered endosomal escape of pore-forming Listeriolysin O toxin-coated gold nanoparticles

**DOI:** 10.1186/s12951-019-0543-6

**Published:** 2019-10-17

**Authors:** Ismael Plaza-GA, Vanesa Manzaneda-González, Matic Kisovec, Víctor Almendro-Vedia, Mónica Muñoz-Úbeda, Gregor Anderluh, Andrés Guerrero-Martínez, Paolo Natale, Iván López Montero

**Affiliations:** 1 Departamento de Química Física, Universidad Complutense de Madrid, Madrid, Spain; 2 Instituto de Investigación Hospital “12 de Octubre” (i+12), Madrid, Spain; 3 Department of Molecular Biology and Nanobiotechnology, National Institute of Chemistry, Ljubljana, Slovenia

**Keywords:** Nanoparticles, Lysosomal escape, Listeriolysin O toxin, Quantum dots, Drug delivery

## Abstract

**Background:**

A major bottleneck in drug delivery is the breakdown and degradation of the delivery system through the endosomal/lysosomal network of the host cell, hampering the correct delivery of the drug of interest. In nature, the bacterial pathogen *Listeria monocytogenes* has developed a strategy to secrete Listeriolysin O (LLO) toxin as a tool to escape the eukaryotic lysosomal system upon infection, allowing it to grow and proliferate unharmed inside the host cell.

**Results:**

As a “proof of concept”, we present here the use of purified His-LLO H311A mutant protein and its conjugation on the surface of gold nanoparticles to promote the lysosomal escape of 40 nm-sized nanoparticles in mouse embryonic fibroblasts. Surface immobilization of LLO was achieved after specific functionalization of the nanoparticles with nitrile acetic acid, enabling the specific binding of histidine-tagged proteins.

**Conclusions:**

Endosomal acidification leads to release of the LLO protein from the nanoparticle surface and its self-assembly into a 300 Å pore that perforates the endosomal/lysosomal membrane, enabling the escape of nanoparticles.

## Background

Drug delivery has become an important research field for the improvement of the safety and efficacy ratio in the application of established and new discovered drugs [1]. One important requisite is the robust vehiculation of the drug with nanocarriers, such as lipoplexes [2], polyplexes [2], liposomes [3], microspheres [4] and spherical or rod-shaped gold-nanoparticles [1, 5, 6]. The use of gold nanoparticles (Au-NPs) for biomedical purposes has considerably increased in the last few years owing to their good bio-compatibility and exceptional optical properties, allowing for the detection of small changes in the dielectric environment surrounding the particles [7, 8]. Among said optical properties, nanostructured metals exhibit a surface plasmon resonance (SPR) signal (from 500 nm to the near-infrared (NIR) [9]) emerging from the interaction between light and the conduction electrons, leading to a characteristic particle color depending on the size, shape, and composition of the NPs [10]. The NIR signal is of special interest for bio-medical applications as cellular tissue barely absorbs light in that wavelength range, thus providing an efficient spectral window for signal detection [11]. Therefore, NPs are not only vehicles but compounds of interest themselves for e.g. plasmonic photothermal therapy (PPTT), where laser-induced heating of Au-NPs induces the apoptosis or thermolysis of cancer cells [5]. To realize the full potential of Au-NPs in cellular applications, challenges regarding their controlled uptake by cells, localization in the cellular cytosol, or directed targeting of organelles must be overcome. The functionalization of the NP surface with engineered coatings improves the hydrophilicity and biocompatibility of Au-NPs, as well as providing a means to attach unique targeting molecules to facilitate the cellular uptake of the carrier and allow the targeted delivery of the drug load [12]. This modification is crucial, as suitable surface functionalization has a great impact on the success or failure of drug delivery. Au-NP internalization by eukaryotic cells occurs via the endosomal pathway, which starts with the invagination of the plasma membrane and eventually produces lysosomes for their degradation [13]. Such Au-NP degradation and/or rejection are major drawbacks and important issues as drug synthesis is expensive and the therapy strategies developed so far are not very efficient [14]. Moreover, NPs have been recently shown to be expelled from the cells after an initial uptake [15]; thus, for therapeutic applications in PPTT, the residence time of the NPs inside the cell needs to be significantly increased.

Here, we present the surface functionalization of Au-NPs with the pore-forming *Listeria monocytogenes* Listeriolysin O (LLO) toxin as a “proof of concept” to promote the lysosomal escape of Au-NPs inside mouse embryonic fibroblasts (MEFs) and reduce or even prevent their expulsion [16]. The LLO belongs to the cholesterol-dependent cytolysin family (CDC) and is produced by the bacterial pathogen *Listeria* inside eukaryotic host cells during infection [17]. LLO facilitates the escape of the bacteria from the lysosomes of the host cell, thus guaranteeing the survival of the pathogen [18, 19]. LLO also plays a critical role in the protective immune response to *L. monocytogenes.* Increasing evidence arises that LLO is a multifunctional virulence factor that elicits a eukaryotic host response independent from the mechanical membrane disruption including cell proliferation, the activation MAP kinases, mucus secretion in intestinal cells or the modulation of cytokine expression in macrophages. More details on these processes are discussed extensively by Vázquez-Boland et al. [20].

**Fig. 1:**
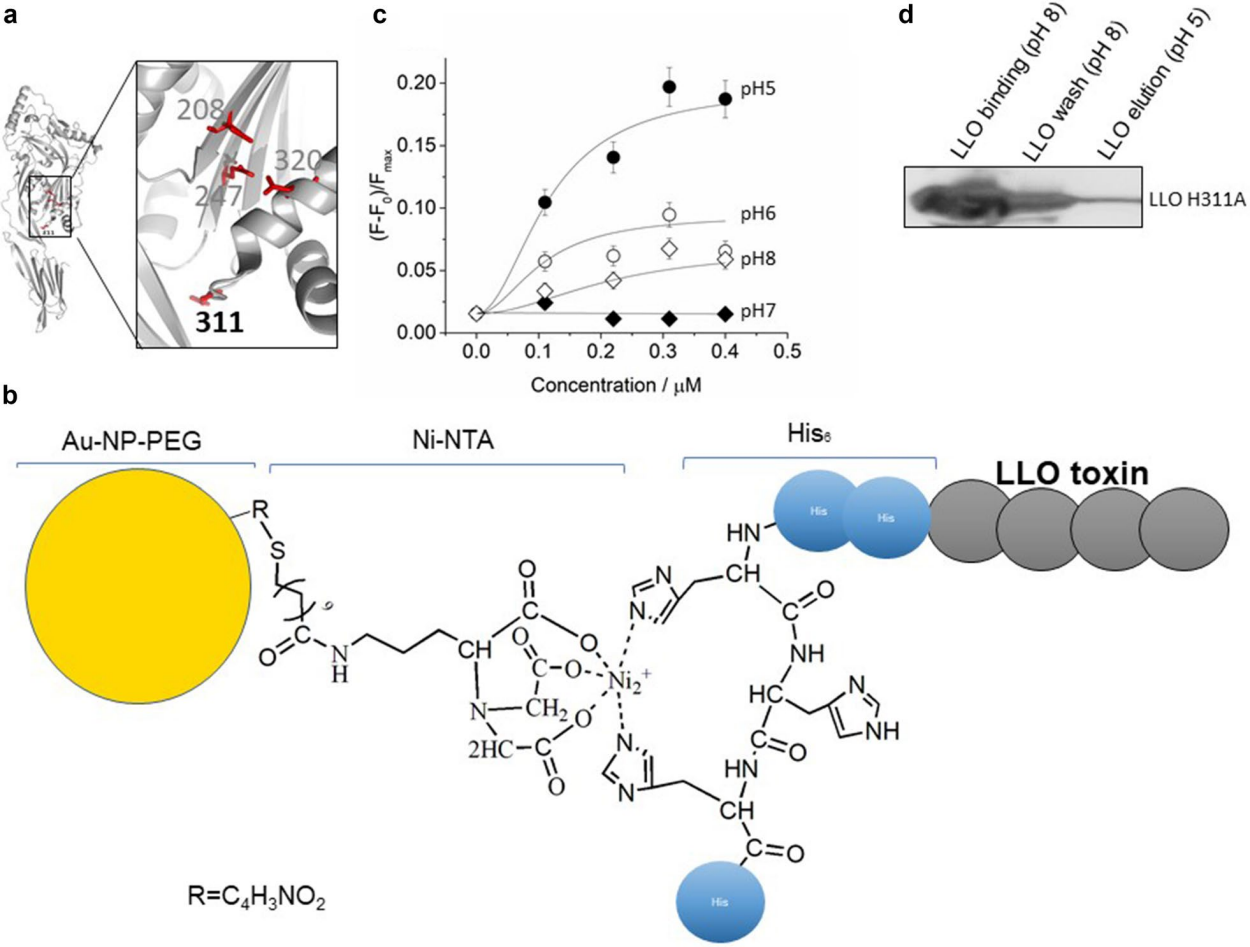
**a** Crystal structure of LLO monomer (pdb:4CDB) highlighting the acidic triad and H311 amino acid residue; **b** scheme of the surface functionalization of Au-NP; **c** In-vitro calcein release experiments in the presence of increasing amounts of His-LLO H311A at pH 5, 6, 7, and 8 at 37 °C; **d** In-vitro binding and release of His-LLO H311A to/from Au-NPs. The supernatant was separated from the Au-NPs by centrifugation, showing the unbound excess of LLO H311A at pH 8 (lane 1), loosely bound His-LLO H311A fraction washed with sodium phosphate buffer at pH 8 (lane 2), and the specifically bound Ni-NTA-bound His-LLO H311A fraction eluted with sodium phosphate buffer at pH 5 (lane 3). His-LLO H311A was visualized by western blotting with monoclonal anti-histidine antibody

Cholesterol-dependent cytolysins are composed of four domains, each having a distinct role in the pore formation. Membrane binding and cholesterol recognition is mediated by the domain 4 [21, 22]. Once bound on the membrane surface, up to 50 monomers self-assemble into a pre-pore with a diameter of approximately 300 Å [23]. Within the CDCs, the ability of LLO toward pore formation is unique, being the most stable in the acidic pH environment, while neutral environment rapidly leads to aggregation of the protein [22, 24]. This allows maximum activity inside the late endosome or lysosomes, where pore formation in the lysosomal membrane eventually destabilizes and breaks the lysosome liberating its contents [22, 25]. The conformational rearrangement of the LLO protein during pore forming process is controlled by the presence of the acidic amino acid triad glutamine 247 (Glu247), asparagine 320 (Asp320), and glutamine 208 (Glu208) (Fig. 1a) [26]. At physiological pH, this acidic triad is deprotonated, and the protein remains in compact conformation burying the hydrophobic amino acids inside the protein core. Upon protonation due to acidification, the acidic triad is destabilized, and this promotes further changes in the conformation of the protein, which ultimately lead to pore formation [24]. Once released into the eukaryotic cytoplasm, *Listeria* proliferates and infects adjacent cells to initiate a new round of infection [19].

## Results and discussion

In this work, we have used a histidine-tagged variant of LLO, where the histidine residue at position 311 is substituted by an alanine residue (LLO H311A mutant; Fig. 1a) [27]. Due to such change in the close vicinity of the acidic triad, the H311A mutation results in an acidity threshold for LLO pore maturation, whereby it only takes place at pH below < 6 [27]. In addition, an amino-terminal histidine tag was employed not only for affinity purification of the protein, but also for surface functionalization of the Au-NPs. We purchased PEGylated Au-NPs (InnovaCoat^®^ GOLD Maleimide; SKU: 270-0005) and further functionalized them with thiolated nitrilotriacetic acid (SH-NTA). This approach allows to specifically attach the histidine-tagged LLO protein to the particle surface (Fig. 1b) and its release in an acidic environment. We tested the pore-forming activity of the purified LLO protein in vitro by the release of liposome-entrapped calcein. Calcein is a fluorescent dye with excitation and emission wavelengths of 495 and 515 nm, respectively. At concentrations above 70 mM, the calcein fluorescence is attenuated by self-quenching, being thus a suitable indicator of lipid vesicle leakage frequently used in pore forming toxins research [22]. We encapsulated calcein in small unilamellar vesicles (SUVs) made of 1-palmitoyl-2-oleoyl-*sn*-glycero-3-phosphocholine and cholesterol (POPC/Chol; 1:1; mol/mol) and monitored the change in the calcein fluorescence signal in the presence of increasing amounts of purified His-LLO H311A or His-LLO *wild type* LLO. In vitro, we observed stimulated pore-formation activity of LLO H311A at pH 5 and moderate activity at pH 6–8 (Fig. 1c), while no strong pH-dependent pore formation was observed in the case of the LLO *wild type* protein (Additional file 1: Figure S1). Although the presence of cholesterol is required for pre-pore formation and acidification triggers the maturation in vivo, additional factors, as the PEST domain (proline (**P**), glutamic acid (**E**), serine (**S**), and threonine (**T**), present in the amino terminal part of the protein, regulate the *Listeria* virulence and the LLO pore-formation activity to selectively occur in late endosomes or lysosomes at low pH [28]. We also tested the integrity of POPC/Chol liposomes during LLO pore formation. We immobilized His-LLO *wt* on a 10 mol% 1,2-dioleoyl-*sn*-glycero-3-[(*N*-(5-amino-1-carboxypentyl) iminodiacetic acid) succinyl (DOGS-NTA)-doped POPC supported lipid bilayer (SLB) at pH 8. Next, giant unilamellar vesicles (GUVs) made of POPC/Chol (PC/Chol-GUVs) were added to the SLB. In the presence of LLO, we observed the attachment of sedimented PC/Chol-GUVs to the SLB layer, as indicated by the reduced GUV mobility on the SLB surface. This local attachment occurs most likely through binding of PC/Chol-GUV to the cholesterol binding motif of the surface LLO protein (Additional file 1: Figure S2A), which was not observed in the absence of LLO (Additional file 1: Figure S2B), where the position of the sedimented PC/Chol-GUVs was found to change during confocal laser scanning microscopy (CLSM) imaging. To visualize the LLO pore formation, we added calcein to the buffer (pH 8). Calcein is membrane-impermeable and no calcein was observed inside the lumen of PC/Chol-GUVs until the buffer pH was acidified from 8 to 5.5. At pH 5.5, the calcein influx into the PC/Chol-GUVs is rather fast, being almost complete in 5 min (Additional file 1: Figure S2A). Acidification of the buffer triggers the surface release of the LLO protein and induces the LLO pore formation in the cholesterol-containing PC/Chol-GUVs. The PC/Chol-GUVs did not collapse at the employed LLO concentration (1,9 nM final concentration) nor significant spontaneous disintegration of the sample was observed at incubation times shorter than 30 min (Additional file 1: Figure S2A). Higher concentrations of LLO (100–500 nM) destroy GUVs and GUV permeability cannot be attributed to discrete LLO pore formation [25]. In the absence of LLO, no calcein influx was observed upon acidification (Additional file 1: Figure S2B).

For the present proof-of-concept, it is important that escape occurs in late endosomes or lysosomes at low pH; therefore, we evaluated the LLO surface release of LLO mutant H311A from LLO-bound Au-NPs. At neutral pH, the H311A-LLO mutant can associate into a membrane-bound pre-pore [29], but will not penetrate and disrupt the membrane. LLO was attached via an amino-terminal histidine tag to the Ni-NTA group of the functionalized Au-NPs. The surface bound protein will only release from the surface upon acidification to low pH which will break the interaction of the Ni-NTA group and the histidine residues of the protein (Fig. 1b). The His-LLO H311A protein was incubated in excess with Au-NPs to saturate the protein content on the particle surface. The excess of unbound was removed by centrifugation to separate and collected the Au-NPs carrying LLO H311A from the unbound LLO H311A protein (Fig. 1d, lane 1). A consecutive wash with sodium phosphate buffer at pH 8, followed by centrifugation, allowed the recovery of the particles (Fig. 1d, lane 2). To release the NTA-bound His-LLO H311A from the particle surface, the washed Au-NP pellet was resuspended in sodium phosphate buffer at pH 5 and incubated for 10 min at RT. The acidic pH promoted the release of the protein from the NTA-group of the Au-NPs, and His-LLO H311A was collected in the supernatant after Au-NP separation by centrifugation (Fig. 1d, lane 3).

To obtain some information on the particle surface charge, size and stability we measure the zeta potential and DLS profile of the functionalized Au-NPs in the absence and presence of LLO H311A. We obtain of − 13.1 ± 3.3 mV and − 2.12 ± 0.46 mV for the functionalized Au-NPs with and without LLO AuNP respectively. The presence of the Ni-NTA group on the Au-NP surface reduces the surface change below the threshold value (> − 30 mV and < 30 mV) expected for a stable Au-NPs suspension [30]. DLS experiments indicate that the bare Au-NPs have a hydrodynamic diameter of 35.5 ± 10.7 nm and Au-NPs carrying LLO H311A a hydrodynamic diameter 67.9 ± 3.97 nm. Bare Au-NPs and LLO-NPs polydispersity index of 0.162 +/- 0.029 and 0.377 +/- 0.029 respectively and therefore we exclude excessive particle aggregation during sample preparation. The Au-NPs with bound LLO H311A protein will be hereafter denoted as LLO-NPs.

**Fig. 2:**
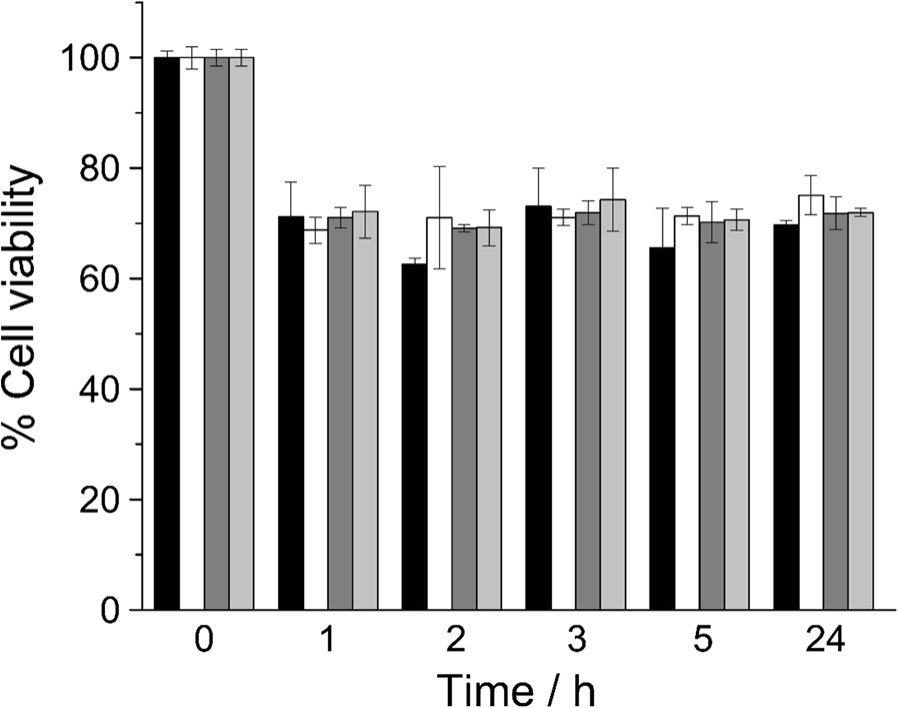
MEF viability in hours after addition of Au-NPs with (black bars) and without (white bars) LLO and Qdots with (dark gray) and without (light gray) LLO. Mean and error were calculated from of 12 replicas per timepoint

The toxicity of LLO-NPs was then evaluated in MEF cultures using the resazurin (7-hydroxy-3H-phenoxazin-3-one-10-oxide)-based alamarBlue^®^ cell viability assay, which reflects the redox state of the cell (fully reduced in live cells and fully oxidized in dead cells) [31]. Figure 2 shows the cell viability results for MEFs in the presence of functionalized Au-NPs (black bars) and LLO-NPs (white bars). For both types of particles, a 30% loss of cell viability was observed within the first 2 h. Beyond that (up to 24 h), no further drop of the cell viability was observed. Therefore, we concluded that LLO-NPs are sufficiently biocompatible with MEF cultures and an easy and straightforward tool to test the LLO-mediated lysosomal escape of Au-NPs.

Lysosomal escape of the functionalized Au-NPs in living MEF cultures was visualized and traced by CLSM; for this purpose, the Au-NPs were replaced by fluorescent quantum dots (Qdots). The employed Qdots are nanometer-sized CdSe/ZnS semiconductor particles (Additional file 1: Figures S3 and S4A) that absorb white light and, depending on their size and specific chemical composition, emit light at shorter (2–3 nm in diameter; blue and green) or longer wavelengths (5–6 nm diameter; orange or red) [32, 33]. We purchased Qdot™ 655 ITK™ Amino (PEG) (~ 8 × 15 nm in size, Thermofisher Scientific) for these experiments, which are coated with an amino polyethylene glycol (PEG-NH_2_) shell extending its size to a hydrodynamic diameter of ~ 20 nm [34] and exhibit a red fluorescence emission maximum at ~ 655 nm [35]. We functionalized these Qdots through the PEG-NH_2_ layer, with a sulfosuccinimidyl 4-(*N*-maleimidomethyl) cyclohexane-1-carboxylate (sulfo-SMCC) cross-linker and thiolated nitrilotriacetic acid (SH-NTA) (Additional file 1: Figure S3), as previously described [36], and first tested their capacity to bind and release His-LLO H311A. As seen for Au-NPs, Qdot-bound LLO H311A was released when the particles were resuspended in phosphate buffer at pH 5 (lane 3; Additional file 1: Figure S4B). If the amount of surface-precipitated LLO H311A is minimal, the Qdots retain about 65% less protein than the Au-NPs. The estimated Au-NP surface (*A*_Au-NPs_ = 5 × 10^4^ nm^2^) is five times larger than that of Qdots (*A*_Qdots_ = 1.2 × 10^4^ nm^2^) and the Au-NPs release 0.051 µM of LLO H311 per nM Au-NP per nm^2^ in vitro in comparison to 0.0175 µM LLO H311 per nM Qdot per nm^2^. This difference may be related to the amount of conjugated PEG-NH_2_ on the commercially available particle in the first place (Fig. 1b). The cell viability of MEFs exposed to Qdots was like that observed for Au-NPs irrespective of the presence of LLO (Fig. 2). The Qdots conjugated with LLO H311A will be hereafter denoted as LLO-Qdots.

**Fig. 3:**
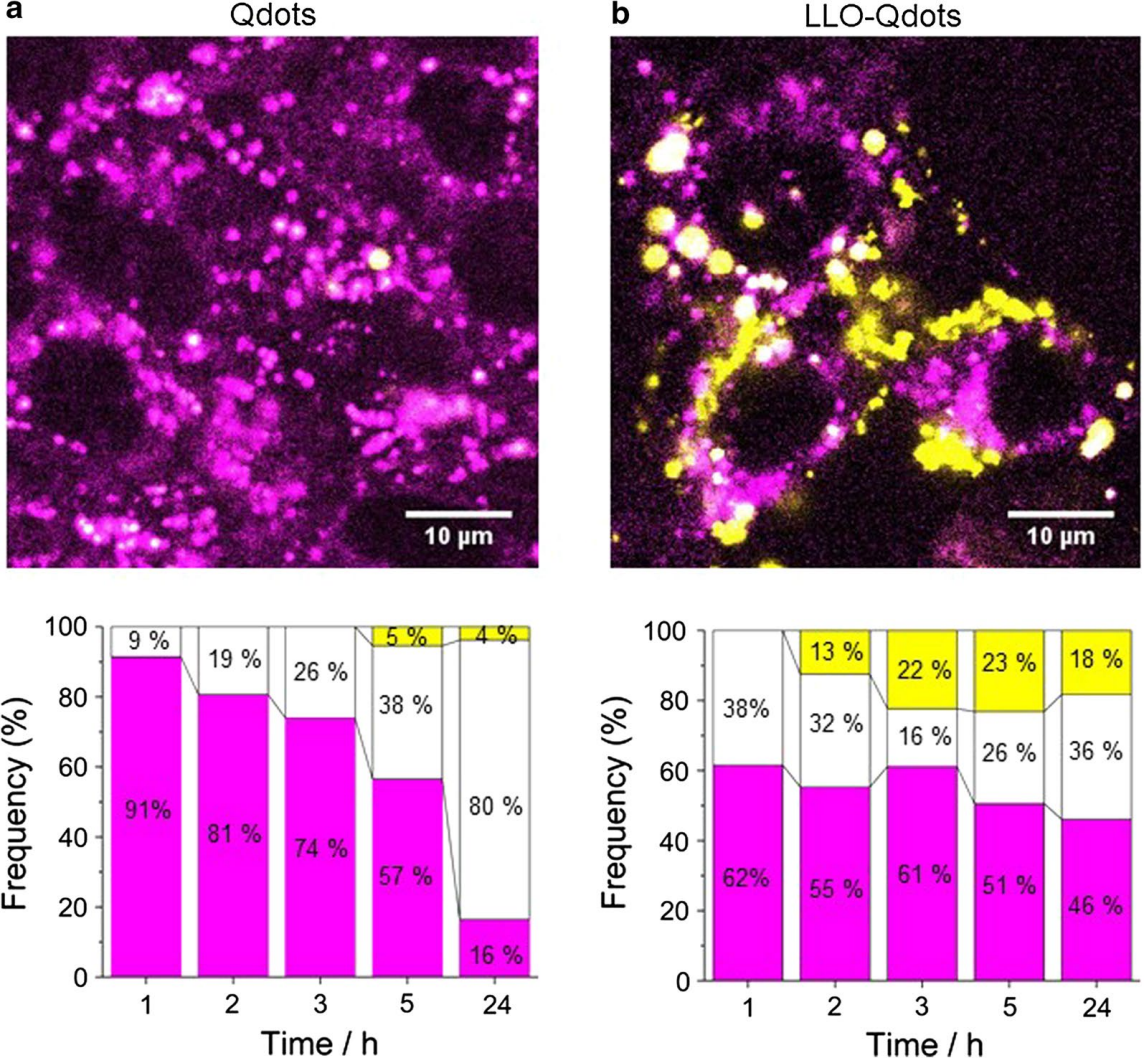
CLSM images of MEFs exposed to bare Qdots (**a**) and LLO-Qdots (**b**). Lysosomes visualized with LysoTracker^®^ Green dye fluorescence at 511 nm (green) and QDot fluorescence at 655 nm (red). Images are presented in false color [48], where the LysoTracker^®^ Green dye is shown in magenta and red Qdots are displayed in yellow when residing in the cytoplasm, or white inside the lysosomal system when colocalized with the magenta LysoTracker^®^ Green dye

Next, MEFs were exposed to LLO-Qdots for analysis of the cellular uptake and eventual lysosomal escape. To distinguish the cytoplasmic or lysosomal localization of the LLO-Qdots throughout the experiments, LysoTracker^®^ Green DND26 dye was employed in addition to Qdots to specifically visualize the cellular lysosomal system (Fig. 3a and b, top images, magenta). As a control, we incubated MEFs with functionalized Qdots without LLO H311A on their surface (bare Qdots, Fig. 3a). The Qdots in the endosome are seen in white color, while the Qdots that have escaped the endosomal system appear in yellow. After 2 h of exposure to LLO-Qdots, we observed, albeit with very low intensity, the presence of yellow spots (Fig. 3b). This means that lysosomal escape of Qdots has occurred to some extent. In general, the overall particle uptake is stronger for LLO-Qdots than for bare Qdots (45% vs 19%). This difference in uptake efficiency may be the result of the presence of a protein corona formed by non-specifically bound LLO protein on the particle surface [37]. When bare Qdots were incubated with 10 mg/mL of BSA previous to the administration to MEFs, the difference in uptake efficiency in comparison to LLO-Qdots was less pronounced (Additional file 1: Figure S5) [37]. At 3 h after LLO-Qdot exposure, the largest number of yellow spots (22%) was observed, which remained almost constant for 24 h (Fig. 3b). To avoid further losses in viability of the MEFs, no experiments were performed at higher initial concentrations of LLO-Qdots. These results demonstrate and validate the LLO toxin-mediated endosomal escape of nanoparticles.

**Fig. 4:**
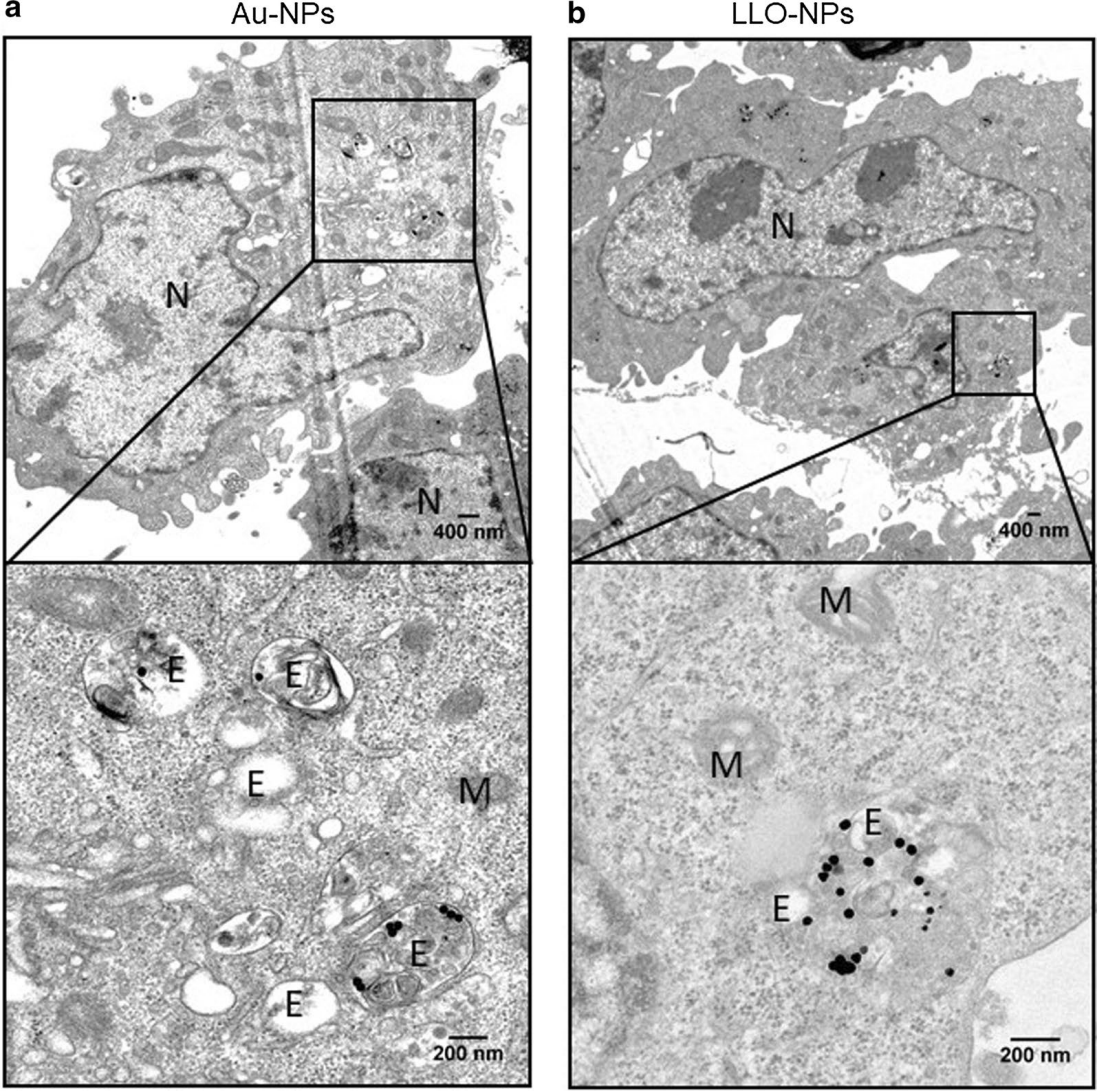
TEM images of MEFs incubated with bare Au-NPs (**a**) and LLO H311A-NPs (**b**). Bottom panels are zoomed areas of the top panel. *N* nucleus, *E* endosomes/lysosomes, *M* mitochondria

As the hydrodynamic diameter of the Qdots is 20 nm with a tendency to cluster into bigger aggregates inside cells (Fig. 3b) or in organic solution [38, 39], we imaged the MEFs by transmission electron microscopy (TEM) after 24 h of incubation with either bare Au-NPs (Fig. 4a), LLO-NPs (Fig. 4b), bare Qdots (Additional file 1: Figure S6C), and LLO-Qdots (Additional file 1: Figure S6D) to obtain a more detailed picture of the particle localization. LLO-NPs and LLO-Qdots were found both inside endosomes/lysosomes and in the cytoplasm (Fig. 4b and Additional file 1: Figure S4D) whereas, in the absence of LLO, the bare particles were solely found inside the endosomes/lysosomes (Fig. 4a and Additional file 1: Figure S4C). We refer to endosome/lysosome as, without further treatment as immunogold labelling, the TEM images do not allow to distinguish between both types of vesicles. In general, as also observed by CLSM, the total amount of bare particles in the cell was smaller (Fig. 4a and Additional file 1: Figure S4C), as the presence of proteins on the particle surface promotes their uptake. Unexpectedly and irrespective of the presence of LLO, the Qdots were always found in the perinuclear area of the MEFs (Additional file 1: Figure S6). Even though Qdots were observed inside the lysosomes, we cannot exclude that, due to their small size, they may also enter the cell via other pathways not involving endosomes [40]. Perinuclear localization was not observed for 40 nm-sized Au-NPs.

Drug-loaded liposomes decorated with the GALA peptide [41] or arginine-rich cell-penetrating peptides (AR-CPPs) [42–44] are the most common tools in the drug delivery field. These peptides induce a remodulation of the lipid bilayer membrane into multilamellar layers, leading to membrane fusion and pore formation. This type of peptides is very versatile, but the tailored design and synthesis of suitable CPPs can be hampered by their lack of target specificity, protease stability, and cytotoxicity [45]. Further, the escape of lipid nanoparticles from acidifying late endosomes or lysosomes has been successfully promoted by surface functionalization with the synthetic octa-arginine peptide (R8) [46, 47]. However, the detailed mechanism of how R8 induces endosomal escape may include the above mentioned CPP-induced liposome fusion, but in general remains elusive and not reliable enough for efficient drug delivery. We thus chose the LLO toxin as it enables better control of the target specificity as the presence of cholesterol in the host membrane allows its application to a wide range of eukaryotic cells [22, 48]. In addition, the use of a histidine-tagged version provides very straightforward and cheap protein production in adequate amounts when compared to solid-phase *tert*-butyloxycarbony (Boc) or fluorenylmethyloxycarbonyl (Fmoc) peptide synthesis [39]. The surface conjugation via Ni^+^-NTA allows the protein release at pH 5.3–4.5, at which the imidazole nitrogen atom of the histidine residue (p*K*_a_ 6.0) becomes protonated that disrupts the coordination between the histidine and transition metal [49]. In the cell, this will only be the case when the Au-NPs reside in sorting endosomes, late endosomes and lysosomes with luminal pH values of 6.0, 5.5, and 4.9, respectively [50]. Up to 30% cytotoxicity was observed during exposure of MEFs to NPs or Qdots. Due to the different size and composition of Au-NPs and Cd/Se Qdots, the strategy of using surface-conjugated LLO to promote escape from the lysosomes highlights the all-round potential of this tool and is therefore an important contribution to the advancement of the delivery of large solid metal nanoparticles for therapeutic applications.

## Conclusion

To our knowledge so far, we provide for the first-time evidence of the lysosomal escape of 40 nm-size gold nanoparticles. These results allow the further development of the delivery strategy by changing either the type of Au-NP (size and geometry) for applications as photothermal therapy or to decorate the surface of the nanoparticle with signaling and targeting proteins able to direct the Au-NPs to G protein-coupled receptors (estrogen and progesterone receptors) of tumor cells or to specific organelles such as mitochondria or Golgi within cancer cells or cells suffering dysfunction related to these organelles.

## Methods

### Functionalization of nanoparticles

InnovaCoat^®^ GOLD nanoparticles (10 nM; Antibody BCN, 270-0015) were incubated with 2 mM of thiolated nitrilotriacetic acid (NTA terminal-SAM formation reagent, Sigma Aldrich 792438) according to manufacturer’s instruction. After conjugation of the NTA group to the Au-NP, the particles were centrifuged for 6 min at 9000*g* and pelleted particles were resuspended in 50 mM phosphate buffer pH supplemented with 1 mM of nickel chloride. Consequently, the AuNPs-NTA-(Ni) particles were centrifuged for 6 min at 9000*g* and pelleted particles were resuspended in 50 mM phosphate buffer pH and stored at 4 °C for further use. Quantum dots 655 (maximum of emission) coated with polyethylene glycol (QD655-PEG; Q21521MP; Invitrogen) were functionalized with nickel nitrilotriacetic acid (Ni-NTA) to allow conjugation of histidine-tagged proteins on their surface [36]. Briefly, 8 µM of QD655-PEG dissolved in borate buffer 50 mM was incubated for 1 h at RT with 80 µM of the sulfosuccinimidyl 4-(*N*-maleimidomethyl) cyclohexane-1-carboxylate (sulfo-SMCC) cross-linker dissolved in DMF. QD655::sulfo-SMCC was pelleted three times by centrifugation for 15 min at 7437*g* in a Beckman microfuge (all centrifugations were performed under the same conditions) to remove non-reacted sulfo-SMCC from the mixture and resuspended in 50 mM PBS. In a second reaction, 800 µM of thiolated nitrilotriacetic acid (NTA terminal-SAM formation reagent, Sigma Aldrich 792438) dissolved in DMSO was added to the QD655-PEG::sulfoSMCC mixture and incubated for 2 h at RT under continuous stirring. QD655-PEG::sulfoSMCC::S-NTA (QD655-NTA) was pelleted three times by centrifugation to remove non-reacted SH-NTA from the mixture and then resuspended in 50 mM PBS. Finally, QD655-NTA was incubated with 800 µM of nickel chloride for 1 h at RT under continuous stirring to coordinate the NTA group for efficient binding to the histidine residue of the protein of interest. QD655-Ni-NTA was pelleted three times by centrifugation to remove the non-reacted nickel from the mixture and resuspended in 50 mM PBS.

### LLO binding to functionalized nanoparticles

The purified His-LLO H311A protein was mixed at different mol/mol ratios with functionalized 1 nM Qdots (1000:1) and 0.2 nM Au-NPs (12,000:1) and tested for protein binding and release. First, Qdots or Au-NPs were incubated with His-LLO for 10 min in 50 mM sodium phosphate buffer pH 7.4 and then centrifuged for 15 min at 7500*g* (Beckman Microfuge 18 Centrifuge) to separate LLO-bound nanoparticles from unbound LLO. The supernatant was collected for Western blot analysis and the pellets were resuspended in upon resuspension in 50 mM sodium phosphate buffer pH 7.4. To wash the LLO-bound NPs, upon resuspension in 50 mM sodium phosphate buffer pH 7.4, the samples were centrifuged for 15 min at 7500*g* (Beckman Microfuge 18 Centrifuge) and the obtained pellets were separated from the supernatant and resuspended in fresh 50 mM sodium phosphate buffer pH 7.4. This was repeated three times before the pellets were finally resuspended in 50 mM sodium phosphate buffer pH 5.5 to release the LLO protein from the particle surface. The sample was centrifuged at 7500*g* and the pellet was separated from the supernatant. To visualize the amount of LLO bound to the NPs, Western blot analysis of the supernatants was performed for the initial binding, final washing, and release steps.

### Confocal laser scanning microscopy

Confocal laser scanning microscopy (CLSM) images were taken at 1, 2, 3, 5, and 24 h to monitor the evolution of the lysosomal escape of Qdots mediated by His-LLO. MEFs were seeded at 1 × 10^4^ cells per cm^2^ in a four-chamber Lab-Tek^®^ slide (Thermofisher) and incubated in complete DMEM for 24 h at 37 °C. To image the lysosomal escape of LLO-conjugated Qdots, MEFs were supplemented with LLO H311A-Qdots or non-conjugated bare Qdots at a final concentration of 1 nM together with 50 nM of LysoTracker^®^ Green DND-26 (Invitrogen) to simultaneously visualize the lysosomes and incubated up to 24 h. The LysoTracker^®^ Green fluorescence was excited at 488 nm and the QDot655 fluorescence at 561 nm. The Lab-Tek^®^ slide was mounted on the temperature-controlled (37 °C) stage of an inverted Nikon Ti-E microscope equipped with a Nikon point scanning confocal microscope module C2, Nikon Plan Apo 100 × NA 1.45 oil immersion objective and two lasers (488 nm and 561 nm; Sapphire laser). Quantification was performed by counting the red (Qdots), green (LysoTracker^®^ Green), and yellow colored signal dots (colocalization). Red and green colors were changed to magenta and yellow producing a white signal to indicate colocalization [51]. The images were captured with the Nikon NIS-Elements software and further processed with ImageJ [52].

### Transmission electron microscopy

For TEM imaging, MEFs were seeded at 1 × 10^4^ cells per cm^2^ in a four-chamber Lab-Tek^®^ slide (Thermofisher) and incubated in complete DMEM for 24 h at 37 °C. To image the endosomal/lysosomal escape of His-LLO H311A-coated Au-NPs and His-LLO H311A-coated Qdots, MEFs were exposed for 24 h to His-LLO H311A-AuNPs, LLO H311A-Qdots, bare Au-NPs, or bare Qdots at a final concentration of 0.2 nM (Au-NPs) or 1 nM (Qdots). The cells were collected by centrifugation for 15 min at 209*g* (Beckman F301.5 Rotor), washed and fixed with 2% glutaraldehyde in PBS buffer, and then stained with 1% osmium tetroxide and 1.5% potassium cyanoferrate. The samples were gradually dehydrated with acetone, embedded in Epon, and cut by ultramicrotomy (60 nm sections) for observation. TEM images were taken with a JEOL JEM-1010 transmission electron microscope operating at an acceleration voltage of 80 kV (CNME, UCM, Spain). The captured images were further processed with the ImageJ software package [47].

## Supplementary information

**Additional file 1.** Supplemental Experimental Section, Supplemental Figures S1 to S6 and Supplemental References.

## Data Availability

All data generated or analyzed during this study are included in this published article.

## Abbreviations

Au-NP: gold nanoparticle
Chol: cholesterol
CLSM: confocal laser scanning microscopy
CDC: cholesterol-dependent cytolysins
DOGS-NTA: 1,2-dioleoyl-sn-glycero-3-[(*N*-(5-amino-1-carboxypentyl) iminodiacetic acid succinyl
DMEM: Dulbecco’s Modified Eagle Medium
DMF: dimethylformamide
GUVs: giant unilamellar vesicles
His: histidine
LLO: Listeriolysin O toxin
MEF: mouse embryonic fibroblasts
Ni-NTA: nickel-nitrilotriacetic acid
NP: nanoparticle
PEG: polyethylene glycol
POPC: 1-palmitoyl-2-oleoyl-sn-glycero-3-phosphocholine
PBS: phosphate buffer saline
Qdots: quantum dots
sulfo-SMCC: sulfosuccinimidyl 4-(*N*-maleimidomethyl) cyclohexane-1-carboxylate
SLB: supported lipid bilayer
SH-NTA: thiolated nitrilotriacetic acid
TEM: transmission electron microscopy
