# Supplementary material for: pH-triggered endosomal escape of pore-forming Listeriolysin O toxin-coated gold nanoparticles
Source: J Nanobiotechnology. 2019 Oct 17;17:108. doi: 10.1186/s12951-019-0543-6 (PMC6798460; doi:10.1186/s12951-019-0543-6)
Supplement: Supplementary file 1 — Additional file 1. Supplemental Experimental Section, Supplemental Figures S1 to S6 and Supplemental References. [file 12951_2019_543_MOESM1_ESM.docx]

Supporting Information

pH-triggered Endosomal Escape of Pore-forming Listerolysin O Toxin-coated Gold Nanoparticles

Ismael Plaza-GA^[a]^, Vanesa Manzaneda-González^[a]^, Matic Kisovec^[c]^, Víctor Almendro-Vedia^[a,b]^, Mónica Muñoz-Úbeda^[b]^, Gregor Anderluh^[c]^, Andrés Guerrero-Martínez^[a]^, Paolo Natale*^[a,b]^ and Iván López Montero*^[a,b ]^

[a] Departamento de Química Física, Universidad Complutense de Madrid, Madrid, Spain.

[b] Instituto de Investigación Hospital "12 de Octubre" (i+12), Madrid, Spain.

[c] Department of Molecular Biology and Nanobiotechnology, National Institute of Chemistry, Ljubljana, Slovenia

Corresponding: [pnatale@ucm.es](mailto:pnatale@ucm.es); [ivanlopez@quim.ucm.es](mailto:ivanlopez@quim.ucm.es)

**Keywords:** nanoparticles • endosomal escape • Listerolysin O toxin • quantum dots • drug delivery

# SUPPLEMENTARY EXPERIMENTAL SECTION

**Protein purification**

To produce LLO heterologously in *Escherichia coli* BL21 (DE3), competent cells were transformed according to standard procedures [1] with plasmid pPROEX-HTb-HIS-LLO WT or pPROEX-HTb-HIS-LLO H311A coding for His-LLO WT or mutant His-LLO H311A, respectively (G. Anderluh, Laboratory Stock). Plasmid-transformed LB (supplemented with 100 μg/mL of ampicillin) grown *E. coli* BL21 (DE3) overnight culture was diluted at 1:100 in fresh LB supplemented with 100 μg/mL ampicillin and grown at 37 ºC in an orbital shaker to an optical density (OD600) of 0.6. LLO production was induced by addition of 0.5 mM isopropyl β-D-1-thiogalactopyranoside (IPTG) and the cells were further grown for additional 2 h. Bacterial cells were collected by centrifugation for 10 min at 10000*g* (rotor TA-10-250; Allegra centrifuge, Beckman coulter) at 4 °C and resuspended in 50 mM sodium phosphate buffer supplemented with 20% sucrose (w/v). The collected cells were flash-frozen in liquid nitrogen and stored at -80 °C until further use.

For purification, the frozen cells were gently thawed and supplemented with 1 mM PMSF (Sigma) and 1 mM DNAaseI (Sigma), and broken by sonication (10 min, 30 sec on/off cycles at 40% amplitude; Sonics Materials™ VCX 500 Ultrasonic Microprocessor). Next, the cell debris and inclusion bodies were pelleted by centrifugation (10 min at 10000*g*; TA-14-50; Allegra centrifuge, Beckman coulter), and the pellet containing inclusion bodies was resuspended in 8 M urea for 15 min at 20 °C to solubilize the LLO inclusion bodies. The non-urea solubilized material was removed by centrifugation (10 min at 10000*g*; TA-14-50; Allegra centrifuge, Beckman coulter) at 20 °C and the supernatant was loaded on sodium phosphate buffer 50mM pH 8 (IMAC equilibration buffer) equilibrated in a 5 mL HiFliQ Ni-NTA FPLC column (Generon). The column was washed with five column volumes of IMAC equilibration buffer, where the first volume was applied very slow at a flow of 0.09 mL/min to allow gentle protein refolding and avoid protein precipitation in the column. Next, the column was washed with five volumes of IMAC equilibration buffer supplemented with 60 mM imidazole to remove unspecific bound proteins and, finally, His-LLO WT or His-LLO H311A were eluted with five volumes of IMAC equilibration buffer supplemented with 300 mM imidazole. In order to concentrate and remove the imidazole, the pooled elution fractions containing His-LLO H311A or His-LLO WT were supplemented with 50 mM Na_2_HPO_4_ to a final pH of 9.5 and then loaded on an equilibrated 5 mL HiTrap Q anion exchange column HP (GE). The column was washed with five volumes of 50 mM sodium phosphate buffer pH 9.5 (IEX buffer) and finally eluted in five column volumes of equilibration buffer supplemented with 1 M KCl and 20% glycerol. The obtained fractions were collected, flash-frozen in liquid nitrogen, and stored at -80 °C until further use. The protein concentration of purified LLO proteins was determined with the Bio-Rad RCDC™ Protein Assay according to the manufacturer’s instructions. SDS-PAGE [2] and Western­blotting techniques were performed according to standard procedures [3] on polyvinylidene difluoride (PVDF; Roche) using monoclonal Anti-polyHistidine−Alkaline Phosphatase antibody produced in mouse (Sigma; A5588) and developed with immobilon chemiluminescent HRP substrate (Millipore) on an ImageQuant LAS 400 system (GE Healthcare).

***In-vitro* calcein release assay**

The pore-forming activity of His-LLO *wt* and His-LLO H311A was evaluated by calcein release assays. For each series, 10 μM calcein-encapsulated SUVs were titrated with increasing amounts of His-LLO *wt* or His-LLO H311A in a final volume of 100 μL of 100 mM sodium phosphate buffer pH 8 at 37 °C. Acidification of the sample was achieved by titration with 1 M HCl to reach a pH of 7, 6, or 5. Calcein fluorescence emission spectra were recorded on an AMINCO-Bowman Series 2 (AB2) scanning spectrofluorometer under excitation wavelength of 480 nm, and recorded from 500 nm to 580 nm with a maximum at 512 nm, slit width of 4 nm, and high Voltage of 500 V. To estimate the amount of released calcein, 1 mM Triton X-100 was added to the sample at the end of each His-LLO H311A titration series to solubilize the remaining intact SUVs and release the total amount of encapsulated calcein. Each data set was normalized with respect to the final maximum fluorescence value obtained upon addition of Triton X-100.

**SUVs**

Preparation of calcein-loaded SUVs and *in-vitro* calcein release assay. Small unilamellar vesicles (SUVs) of either 1-palmitoyl-2-oleoyl-*sn*-glycero-3-phosphocholine (POPC) or POPC and cholesterol (Chol) (1:1 mol) (Avanti) were prepared to test the pore-forming activity of His-LLO. Chloroform-dissolved lipids were mixed at a concentration of 0.1 mM and the solvent was evaporated for 15 min at 60 °C in a vacuum concentrator (Vacufuge plus, Eppendorf). The obtained lipid films were incubated with 80 mM of calcein (Sigma) and sonicated in a bath sonicator (Clifton™ Heated Timed Ultrasonic Baths) for 60 min. The lipid film hydrated and encapsulated the calcein into SUVs of approximately 200 nm. To remove the non-encapsulated calcein, the SUVs were washed three times with milliQ water and centrifuged for 30 min at 200000*g* (TLA 100 rotor; Beckmann Optima MAX-XP ultracentrifuge).

**Supported Lipid Bilayer formation**

For the formation of a supported lipid bilayer (SLB), the surface of a thoroughly cleaned glass coverslip (Menzel-Gläser; 75 × 24 mm) was reduced to a diameter of 6 mm by gluing a 0.2 mL Eppendorf tube upside-down on the glass cover slip surface. The tip of the 0.2 mL Eppendorf tube was then carefully removed and the exposed glass surface inside the tube (chamber) boundaries was treated with 10% (3-aminopropyl) trimethoxysilane dissolved in PBS for 1 hr at RT. Trimethoxysilane interferes with the lipid bilayer adhesion on the charged surface and reduces the membrane rigidity induced by strong adhesion to a charged glass surface. Next, the excess of silane was removed carefully but thoroughly from the reaction chamber by washing the glass surface 5–10 times with PBS. The formation of an SLB was achieved by dilution of a SUV suspension (1 mg/mL POPC/5 mol% DOGS-NTA/ 0.5 mol% rhodamine (Rh)-PE) to reach a final concentration of 0.816 mg/mL of POPC in 20 mM HEPES pH 8, 90 mM KCl. The formation of a planar lipid bilayer was induced by the addition of CaCl_2_ to a final concentration of 100 mM and incubated for 16 h at 4 °C in a hermetic incubation chamber with a saturated sodium chloride solution. CaCl_2_ mediates the interaction between the negative glass surface and membranes, resulting in the formation of a supported membrane bilayer spread on the glass/silane surface. Finally, the formed SLB was washed up to five times with 20 mM HEPES pH 8, 90 mM KCl to remove any small surface-attached vesicles produced during bilayer formation and used for further experiments.

**Electroformation of GUVs**

A chloroform-dissolved lipid mixture (10 μL, 1 mg/mL POPC/ 30% cholesterol/ 2% NBD-PC/ 5% Rh-PE (mol/mol/mol)) was spread in two drops on two conducting indium tin oxide (ITO)-coated glass slides (7.5 cm × 2.5 cm; 15 × 25 Ω/sq surface resistivity) and allowed to dry by solvent evaporation. To build the electroformation chamber, the conductive ITO slides were spaced 1 mm apart using adhesive flexible polyester tape and on top of each spacer was placed a copper adhesive strip to act as an electrode to which the crocodile clips were connected. The chamber was sealed with a water-resistant adhesive putty surrounding the dried lipid film and both ITO slides were held together with binder clips. Once the chamber was formed, the dried lipid film was rehydrated with 200 mM sucrose. Giant unilamellar vesicles were grown by connecting the electroformation chamber to a function generator (10 Hz, ∼1 V) for 16 h.

**Cell culture and viability**

Mouse embryonic fibroblasts (3T3NIH MEF; purchased from Sigma) were cultured in complete high-glucose Dulbecco Modified Eagle Medium (‎DMEM, 25 mM glucose) supplemented with 10% fetal bovine serum (South Africa S1300; Biowest, Nuallé, France), penicillin/streptomycin (final concentration 100 U/mL of penicillin and 100 µg/mL of streptomycin, respectively), and 1% of non-essential amino acids (all Gibco). The cells were grown in a humidified incubator (Forma Steri-Cycle Themofisher; 5% CO_2_) at 37 °C and maintained at a split ratio of 1:10 at 80% of confluence in T75 flasks (Nunc). The cell viability of MEFs was evaluated by the alamarBlue® [4] viability assay (Serotec, Oxon, UK) according to the manufacturer’s instructions. MEFs were lifted and seeded in 96-well plates in a final volume of 100 μL at a density of 1 × 10^3^ cells. After 24 h of incubation at 37 °C with 5% CO_2_ and 95% humidity in the cell incubator, 10 μL of the alamarBlue® reagent was added to each well and incubated for additional 2 h at 37 °C with 5% CO_2_ and 95% humidity. After incubation, the absorption of the individual plates was measured at 570 nm and 620 nm in a [Multiskan™ FC](https://www.thermofisher.com/order/catalog/product/51119000?SID=srch-srp-51119000)  plate reader (Thermo Scientific™), and the cell viability was determined by comparison with non-treated control MEFs according to:

$Cellviability\left( \text{\%} \right)=\frac{\left( \varepsilon_{ox} \right)\lambda_{2}A\lambda_{1}-\left( \varepsilon_{ox} \right)\lambda_{1}A\lambda_{2}of test agent dilution}{\left( \varepsilon_{ox} \right)\lambda_{2}A\lambda_{1}-\left( \varepsilon_{ox} \right)\lambda_{1}A\lambda_{2}of untreated positive growth control of test agent dilution}\times100 (1)$

with *Aλ*_1_ and *Aλ*_2_ as the sample absorption at 570 and 620 nm, respectively, *ε*_620_ox = 34798 M^-1^ cm^-1^ and *ε*_570_ox = 80586 M^-1^ cm^-1^, and *ε*_620_red = 5494 M^-1^ cm^-1^ and *ε*_570_red = 155667 M^-1^ cm^-1^.

**Zeta potential measurements and Dynamic Light Scattering (DLS)**

Au-particle size and zeta potential of 5 pM of bare or LLO coated Au-NPs in 50 mM PBS at a final volume of 1.5 ml were measured at 25 ºC using a phase analysis light scattering technique (Zeta Plus, Brookhaven Instrum. Corp., USA)[5].

LLO H311A was bound to Au-NPs as described (see LLO binding to functionalized nanoparticles in main text). Each electrophoretic mobility datum was taken as an average over 50 independent measurements. Zeta potential (ζ) was obtained from the electrophoretic mobility (µ_e_) using the Henry equation:

ζ = (3ημ_e_)/(2ε_0_ ε_r_ f(κ_D_ a)) (2)

where *η* is the water viscosity (8.904x10-4 N m^-2^ s at 298.15 K); ε_0_ and ε_r_ are the vacuum and relative permittivity (8.854x10-12 J-1 C^2^ m^-1^ and 78.5, respectively); and f (κ_D_ a) the Henry function which depends on the reciprocal Debye length (κ_D_) and the hydrodynamic particle radius (a). For medium-to-large particles in a medium of moderate ionic strength (a >> κ_D_-1), f(κ_D_ a) is assumed as 1.5 following the Smoluchowski approximation [6].

The polydispersity index (PDI) is a measure of particle aggregation and was automatically obtained during measurements of the zeta potentials (ZetaPlus, Brookhaven Instrum. Corp., USA). 50 measurements of duplicates of Au-NPs and LLO-Au-NPs resulted in a PDI of 0,162 +/- 0,029 and 0,377 +/- 0,029. PDI values range from 0 to 1 and indicate nearly monodisperse sample (0-0.08), mid-range values (0.08 to 0.7) or very broad distribution (0.7-1) of particle sizes. For pharmaceutical nanoparticles the desired PDI is < 0.3 [7] therefore we consider LLO-NPs to be fairly monodisperse.

**SUPPLEMENTARY FIGURES**


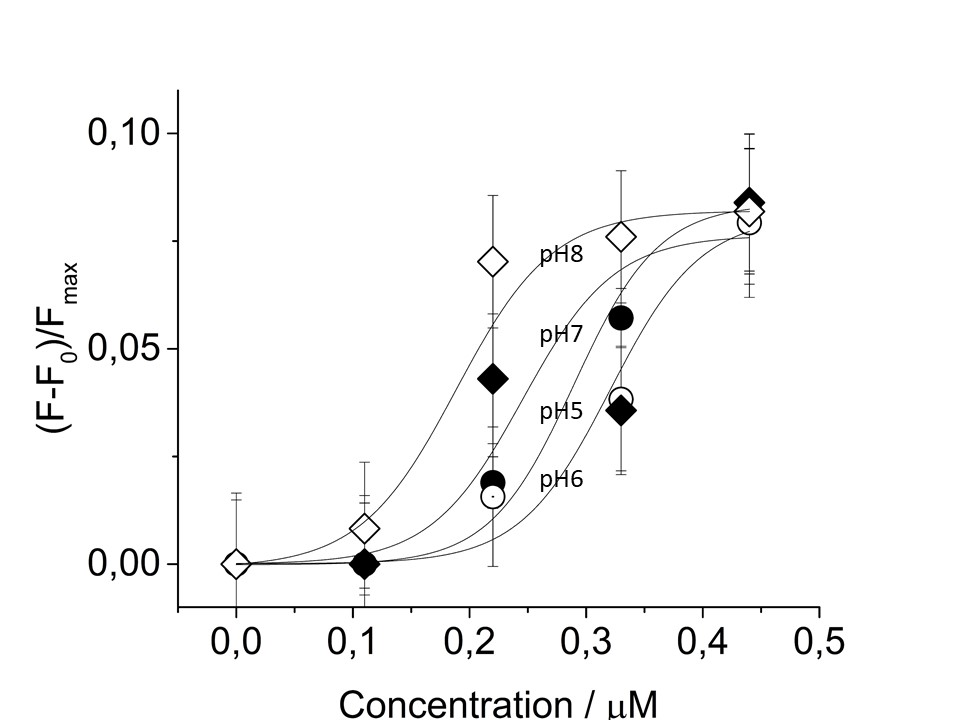


**Figure S1** *In-vitro* calcein release experiments in the presence of increasing amounts of His-LLO *wt* at pH 5, 6, 7, and 8 at 37 °C

.


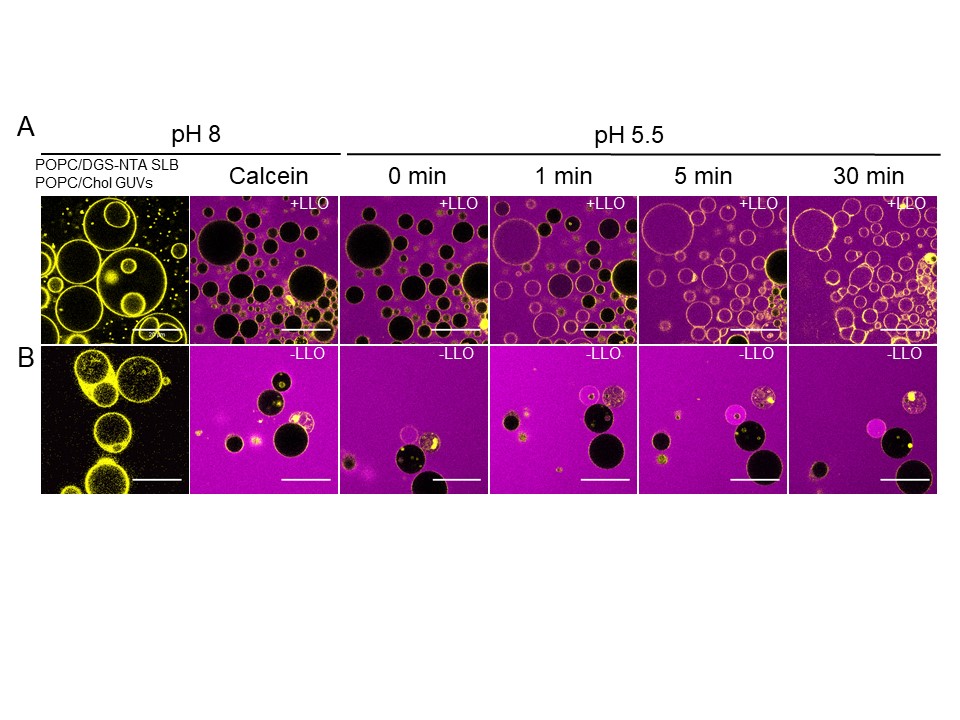


**Figure S2** CLSM imaging of LLO pore formation His LLO *wt* immobilized on SLB (A)His-LLO *wt* SLB in the presence of PC/Chol-GUVs and calcein at pH 8 and 5.5 and (B) SLBs without His LLO *wt* (Control) in the presence of PC/Chol-GUVs and calcein at pH 8 and 5.5. Acidification of the buffer triggers the surface release of the LLO protein and induces the LLO pore formation in the cholesterol-containing PC/Chol-GUVs. The images are presented in false color [8], where the calcein green is shown in magenta and the membrane Rh-PE stain in yellow. Scale bar: 20 μm.


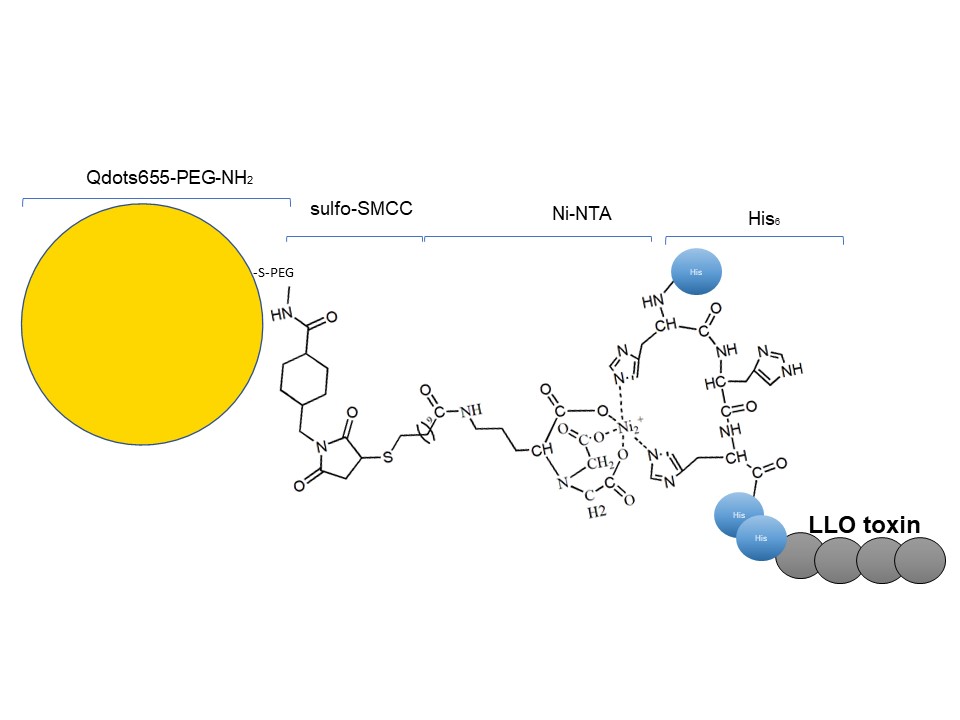


**Figure S3** Scheme of the surface functionalization of Qdots 655-PEG


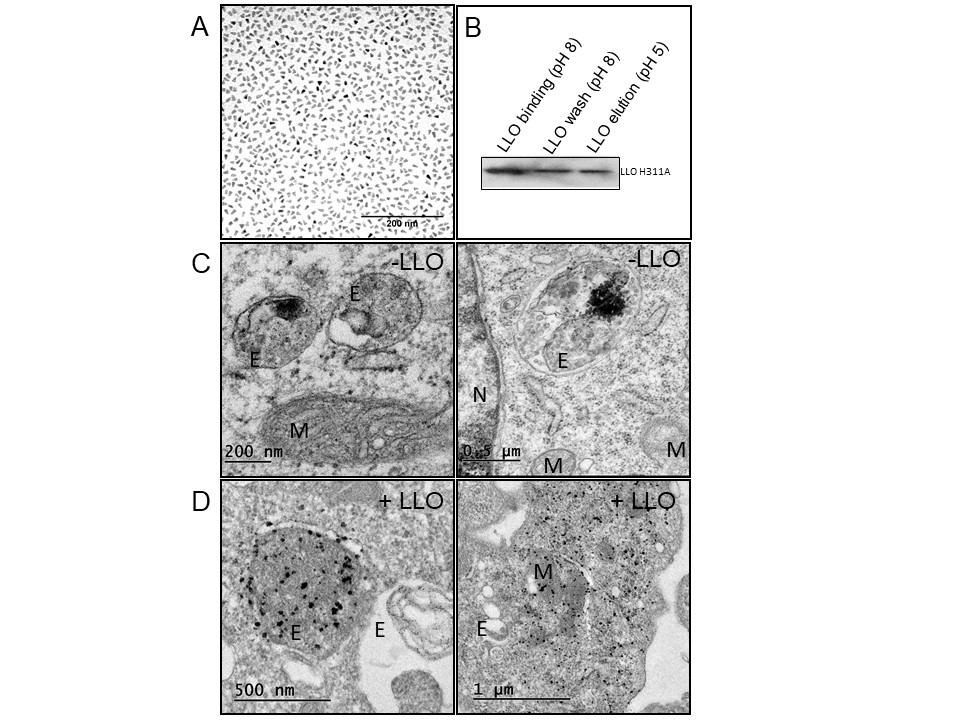


**Figure S4** Qdots used for LLO-mediated endosomal escape in MEFs. (A) TEM image of commercial Qdots-PEG-NH_2_, 655 nm (B) *In-vitro* binding and release of His-LLO H311A to/from Qdots. The supernatant was separated from the Qdots by centrifugation, showing the unbound excess of LLO H311A at pH 8 (lane 1), the loosely bound His-LLO H311A fraction washed with sodium phosphate buffer at pH 8 (lane 2), and the specifically bound Ni-NTA-bound His-LLO H311A fraction eluted with sodium phosphate buffer at pH 5 (lane 3). His-LLO H311A was visualized by western blotting with monoclonal anti-Histidine antibody to test its capacity to bind and release His-LLO. TEM images of MEFs exposed for 24 h to (C) bare Qdots and (D) LLO-Qdots. E: endosomes/Lysosomes, M: mitochondria and N: nucleus


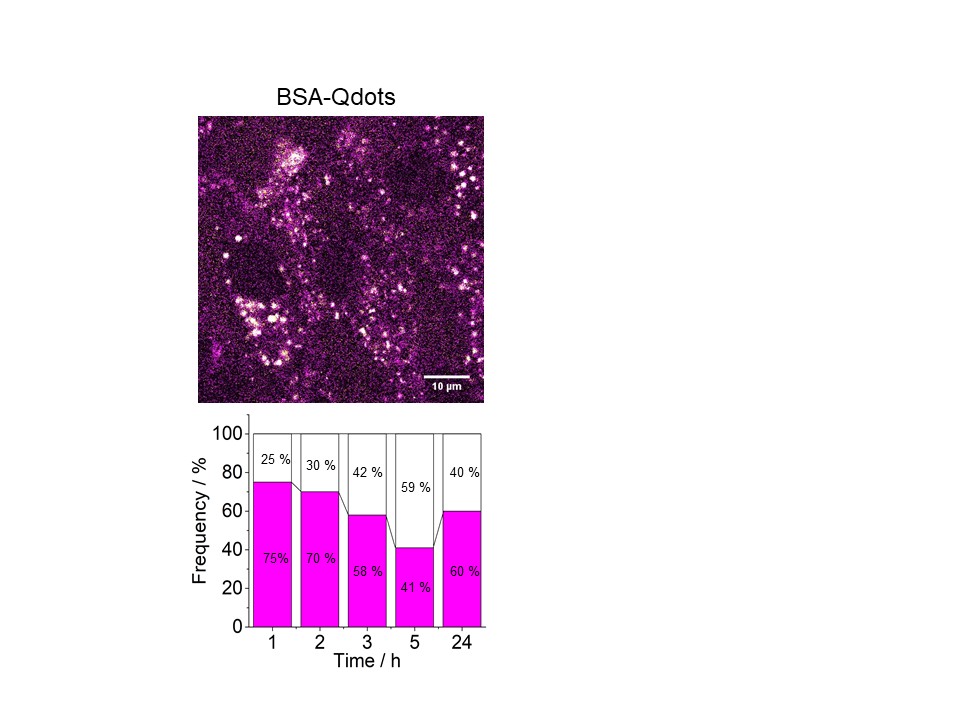


**Figure S5.** CLSM images of MEFs exposed to bare Qdots previously incubated with 10 mg/ml BSA. The lysosomal system was visualized with LysoTracker® Green dye fluorescence at 511 nm (green) and the QDot fluorescence at 655 nm (red). Images are presented in false color [48], where the LysoTracker® Green dye is shown in magenta and red Qdots are displayed in yellow when residing in the cytoplasm, or white inside the lysosomal system when colocalized with the magenta LysoTracker® Green dye.


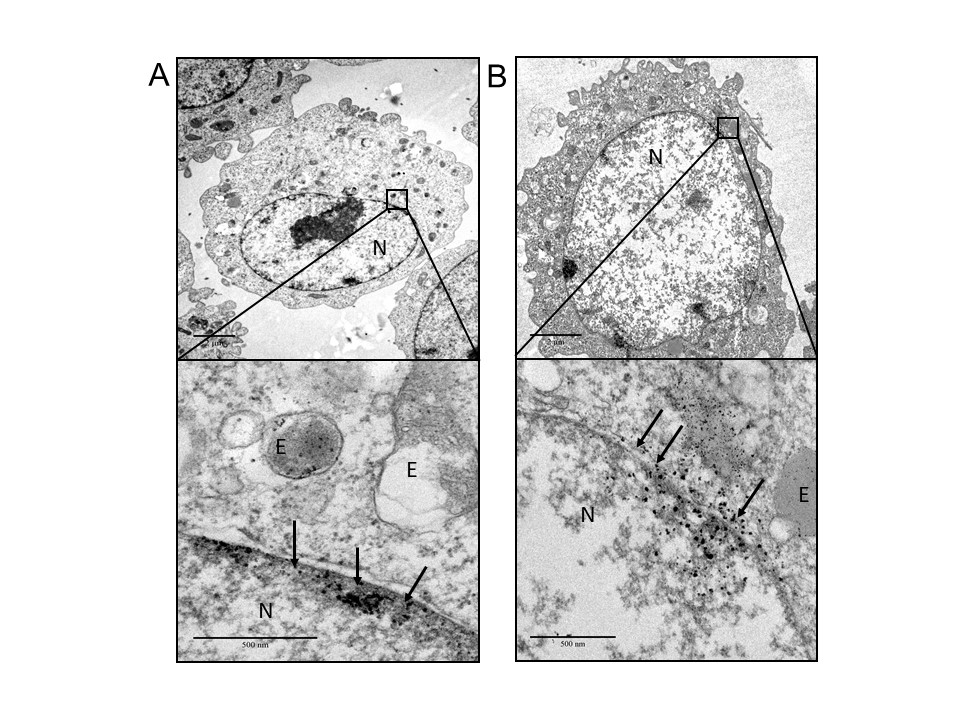


**Figure S6.** TEM images of MEFs incubated with bare Qdots (A) and LLO-Qdots (B). Bottom panels are zoomed areas of the top panel. Arrows indicate the perinuclear area. E: endosomes and N: nucleus

**SUPPLEMENTARY REFERENCES**

1. Sambrook J: *Molecular cloning : a laboratory manual.* Third edition. Cold Spring Harbor, N.Y. : Cold Spring Harbor Laboratory Press, [2001] ©2001; 2001.

2. Laemmli UK: **Cleavage of structural proteins during the assembly of the head of bacteriophage T4.** *Nature* 1970, **227:**680-685.

3. Towbin H, Staehelin T, Gordon J: **Electrophoretic transfer of proteins from polyacrylamide gels to nitrocellulose sheets: procedure and some applications.** *Proc Natl Acad Sci U S A* 1979, **76:**4350-4354.

4. Nociari MM, Shalev A, Benias P, Russo C: **A novel one-step, highly sensitive fluorometric assay to evaluate cell-mediated cytotoxicity.** *J Immunol Methods* 1998, **213:**157-167.

5. Munoz-Ubeda M, Misra SK, Barran-Berdon AL, Aicart-Ramos C, Sierra MB, Biswas J, Kondaiah P, Junquera E, Bhattacharya S, Aicart E: **Why is less cationic lipid required to prepare lipoplexes from plasmid DNA than linear DNA in gene therapy?** *J Am Chem Soc* 2011, **133:**18014-18017.

6. Delgado AV: *Interfacial Electrokinetics and Electrophoresis.* Taylor & Francis; 2001.

7. Danaei M, Dehghankhold M, Ataei S, Davarani F, Javanmard R, Dokhani A, Khorasani S, Mozafari M: **Impact of Particle Size and Polydispersity Index on the Clinical Applications of Lipidic Nanocarrier Systems.** *Pharmaceutics* 2018, **10**.

8. Wong B: **Color blindness.** *Nat Methods* 2011, **8:**441.
